# First Case of COVID-19 Treated with Monoclonal Anti-Spike Antibodies in a Patient with Cystic Fibrosis in Romania

**DOI:** 10.3390/diagnostics12010137

**Published:** 2022-01-07

**Authors:** Iustina Violeta Stan, Victor Daniel Miron, Ioana Alexandra Vangheli, Radu Marian Gheorghiu, Anca Streinu-Cercel, Oana Săndulescu, Mihai Craiu

**Affiliations:** 1 National Institute for Mother and Child Health “Alessandrescu-Rusescu”, 020395 Bucharest, Romania; iustinas@yahoo.com (I.V.S.); vadanaioana@yahoo.ro (I.A.V.); radu.rgm@gmail.com (R.M.G.); mihai.craiu@umfcd.ro (M.C.); 2 Carol Davila University of Medicine and Pharmacy, 050474 Bucharest, Romania; anca_sc@yahoo.com (A.S.-C.); oana.sandulescu@umfcd.ro (O.S.); 3 National Institute for Infectious Diseases “Prof. Dr. Matei Balș”, 021105 Bucharest, Romania

**Keywords:** cystic fibrosis, COVID-19, monoclonal anti-spike antibodies, casirivimab, imdevimab

## Abstract

Patients with chronic lung conditions, including cystic fibrosis, may be prone to severe COVID-19. Therefore, therapeutic intervention should be prompt and tailored to all associated comorbidities. We report the case of a 17-year-old male adolescent with cystic fibrosis and multiple chronic conditions (bronchiectasis, exocrine pancreatic insufficiency, chronic multidrug resistant *Pseudomonas aeruginosa* colonization, nasal polyposis, chronic sinusitis, ventricular extrasystoles and multiple drug allergies), who presented with an acute episode of productive cough, and was confirmed with moderate COVID-19 based on positive RT-PCR for SARS-CoV-2 and lung imaging showing isolated foci of interstitial pneumonia. Intravenous treatment with the monoclonal antibody cocktail casirivimab and imdevimab was administered. The evolution was favorable, with rapid remission of the inflammatory syndrome and gradual decrease of cough, without progression to severe or critical COVID-19, but with complications such as repeated hemoptysis, which was due to the patient’s underlying conditions, and which required close monitoring for timely adjustment of the patient’s chronic treatment.

## 1. Introduction

Infection with severe acute respiratory syndrome coronavirus 2 (SARS-CoV-2) can lead to a wide range of clinical presentations, from asymptomatic infection to clinically significant pneumonia with varying degrees of severity. Underlying comorbidities are some of the well-characterized factors putting the patient at risk for progression to severe coronavirus disease 2019 (COVID-19). Among these, chronic lung disease has been intensely studied, as viral lower respiratory tract infections may be more severe in this patient population. However, not all chronic lung diseases have been associated with a higher risk of severe COVID-19, and the degree of risk generally appears to depend on the extent of pre-existing lung impairment.

For patients with cystic fibrosis (CF), less information is available in field literature, and some studies point to an overall lower incidence of SARS-CoV-2 infection in this patient population [1,2], but a higher rate of hospitalization among them [3]. Furthermore, once infection does occur, the patient is considered to be at risk of severe COVID-19 and this risk may be dependent on the underlying degree of lung damage, and also the degree of pancreatic or liver damage [4].

In Romania the prevalence of CF is approximately 0.106/10,000 [5], but there is no screening for this disease and many cases are diagnosed late. At the time of this case report, Romania was just experiencing its fourth wave of the COVID-19 pandemic, which started in September 2021 and peaked in October 2021, cumulating a record number of confirmed cases for our country since the onset of the pandemic so far.

We report the first case of COVID-19 treated with monoclonal anti-spike antibodies (mAbs) in a patient with cystic fibrosis in Romania in early November 2021, shortly after the introduction of mAbs in clinical practice in the country.

## 2. Case Report

**Figure 1:**
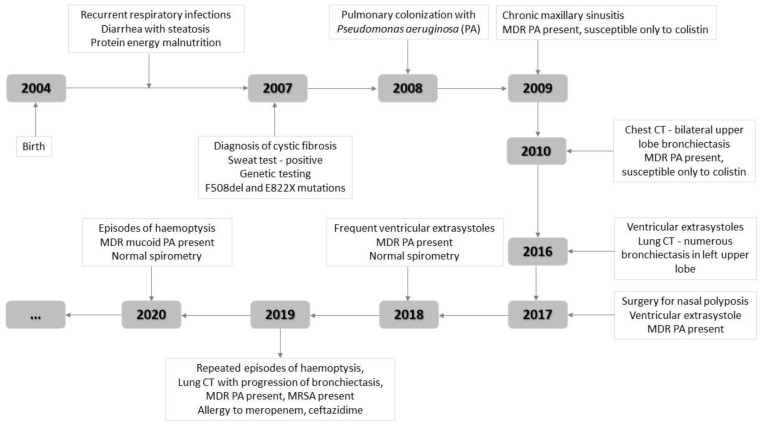
Patient history—the main significant elements for the case. MDR—multidrug resistant, PA—*Pseudomonas aeruginosa*, MRSA—methicillin-resistant *Staphylococcus aureus*, CT—computed tomography.

A 17-year-old male adolescent presented for productive cough with 6-day onset for which he had received, on the general practitioner’s indication, amoxicillin/clavulanate 1 g every 12 h orally at home (for 2 days), followed by cefuroxime 500 mg twice daily orally (for 4 days) and dexamethasone (8 mg/day for 2 days, then 4 mg/day for 2 days, orally), with unfavorable evolution and association of muco-purulent sputum. The patient was known to have CF diagnosed at the age of 3 years (positive sweat test, positive genetic testing for F508del and E822X mutations), malnutrition, bronchiectasis with recurrent hemoptysis, chronic pulmonary colonization with multidrug resistant (MDR) *Pseudomonas aeruginosa* (*P. aeruginosa*) (since the age of 4 years), nasal polyposis (for which he underwent surgery at 13 years), chronic sinusitis, exocrine pancreatic insufficiency, ventricular extrasystoles, allergy to meropenem and ceftazidime, and contraindication to piperacillin/tazobactam, antihistamines, macrolides and salbutamol due to risk of arrhythmia and QT prolongation (Figure 1). The patient had not been vaccinated against COVID-19.

At the initial assessment the patient was clinically stable, with a body mass index (BMI) of 17.9 kg/m^2^ (BMI-for-age percentile < 5), afebrile (36.3 °C), with productive cough and muco-purulent sputum, but respiratory sounds were normal, and peripheral oxygen saturation (SpO_2_) was 98% in ambient air. A multiplex PCR respiratory panel was performed, and it identified SARS-CoV-2, ruling out other concomitant respiratory pathogens. Laboratory investigations showed normal white blood cells (WBCs) count (10.3 × 10^3^/μL), no changes in leukocyte formula, the presence of mild inflammatory syndrome [C-reactive protein = 1.23 mg/dL (normal range < 5 mg/dL), fibrinogen = 432 mg/dL (normal range: 160–390 mg/dL), increased IL-6 = 14.55 pg/mL (normal range < 7 pg/mL)] and negative baseline serology for SARS-CoV-2 (IgM and IgG negative) (Table 1).

**Figure 2:**
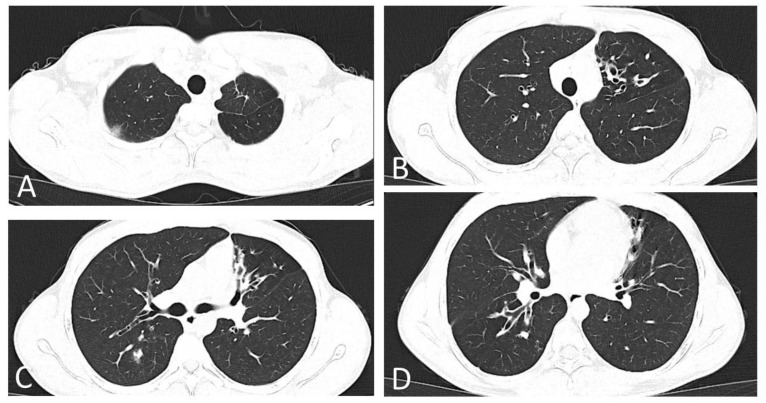
Native chest CT images on the sixth day of disease. (**A**) Native chest CT scan, lung window, apical section: subpleural ground glass opacity in the dorsal segment of the right upper lobe, suggestive for COVID-19. Linear densifications in the anterior segment of the left upper lobe, suggestive for fibrotic sequelae due to the patient’s underlying disease. (**B**) Native chest CT scan, lung window, aortic cross section: clustered cylindrical bronchiectasis in the lingula. (**C**) Native chest CT scan, lung window, hilum section: multiple cylindrical bronchiectasis in the lingula and middle lobe. Pseudonodular condensation area, with ground glass halo, in the right Fowler segment. (**D**) Native chest CT scan, lung window, infrahilar section: multiple cylindrical bronchiectasis in the right Fowler segment, in the anterior segment of the left upper lobe and the superior lingular segment. Pseudonodular condensation area, in the right Fowler segment.

Immediately after confirmation of COVID-19, the patient was referred to Infectious Diseases for evaluation and staging of the disease. At the time of assessment, the patient was afebrile (36.6 °C), with blood pressure 121/78 mmHg, heart rate 85 bpm, respiratory rate 20 breaths/min and SpO_2_ = 97% in ambient air. The electrocardiogram showed no pathological changes (sinus rhythm, PR interval = 138 ms, QRS duration = 76 ms, QT/QTc = 354/421 ms). A native computed tomography of the chest was performed, and it revealed isolated central and peripheral ground glass opacities distributed bilaterally, suggestive for mild COVID-19 pneumonia, on a background of bilateral bronchiectasis and fibrotic-like densities in the left lung apex (Figure 2 and Supplementary Materials).

The patient received treatment with casirivimab 1200 mg and imdevimab 1200 mg administered together as a single 1-h intravenous infusion. The infusion was well tolerated, with no adverse events occurring during administration or in the post-infusion monitoring period.

After receiving anti-spike monoclonal antibodies, the patient was transferred back to the pediatric service for medical supervision. Considering the patient’s chronic conditions, antimicrobial therapy was initiated with colistin (60,000 IU/kg/day, t.i.d) for 14 days, fluticasone propionate in wet nebulization, while also continuing the patient’s chronic treatment (ipratropium bromide inhaler, alpha-dornase-wet nebulization, colistin-wet nebulization, oral pancreatin, oral fat-soluble vitamins (A, D, E, K vitamins), mupirocin nasal ointment).

Three days after admission (on day 9 of illness) the patient presented scant hemoptysis, for which reason fluticasone, colistin and alpha-dornase were stopped and oral tranexamic acid and adrenaline 1‰ in wet nebulization were introduced for 5 days, with subsequent resumption of inhaled therapy with colistin and alpha-dornase. As the patient showed worsening productive cough with persistent muco-purulent sputum 9 days after admission (day 15 of illness) amoxicillin/clavulanate 1 g every 12 h orally was also added to the antibiotic therapy for 7 days.

The evolution of the case was favorable, the patient did not require oxygen administration during hospitalization, with resolution of cough, and complete remission of hemoptysis and sputum production. The duration of hospitalization was 27 days due to the patient’s poor socio-economic condition, but also due to the family’s anxiety. The patient was discharged with persistent positive PCR for SARS-CoV-2 with recommendation to continue chronic treatment (Table 2) for his comorbidities and to come back for periodic evaluation. Seven days after discharge the patient was contacted by phone, and he confirmed having a good general condition with absence of cough and other acute symptoms.

## 3. Discussion

Since the beginning of the COVID-19 pandemic, the entire medical world has been on a steep learning curve to better understand the epidemiology and the pathophysiology of the disease. Living guidelines for the management of COVID-19 have been developed and designed to include up to date information as soon as it becomes available from clinical research. The first clinical trials for treatment options in COVID-19 were started in 2020, initially with repurposed drugs, and later on with specifically designed compounds. Among these, a large body of research has focused on SARS-CoV-2 mAbs. These bind directly to the virus’ spike protein, blocking its interaction with cellular receptors [6] and thereby stopping further viral replication and the development of pneumonia, if administered early, in patients with mild COVID-19 [7]. If administered after pneumonia has occurred, but still within the on-label 7-day interval since symptom onset, in patients with moderate disease, they have been shown to prevent progression to a severe form of COVID-19 [6,7].

In mid-October 2021, during the very peak of the fourth COVID-19 wave in Romania, mAbs were introduced into clinical practice based on the European Medicines Agency’s (EMA) emergency use authorization, for patients at high risk of progressing to severe COVID-19 [8]. We have reported the first administration of mAbs in Romania in a patient with CF who had moderate COVID-19 at the time of initial evaluation. He received the casirivimab/imdevimab antibody cocktail on the sixth day of illness, with a subsequent favorable clinical and laboratory evolution. By late November 2021, two types of mAbs, the casirivimab/imdevimab cocktail and regdanvimab were fully approved by EMA and received a recommendation for marketing authorization in the European Union.

This adolescent with CF had numerous associated chronic conditions (bronchiectasis with recurrent hemoptysis, chronic colonization with MDR-*P. aeruginosa*, pancreatic involvement, chronic sinusitis, ventricular extrasystoles, allergy to meropenem and ceftazidime) which in case of a possible unfavorable evolution due to COVID-19 would have posed serious therapeutic management difficulties. Therefore, immediately after confirmation of SARS-CoV-2 infection, a rigorous analysis of the case was performed by collaboration of pediatricians and infectious diseases physicians and the decision was taken to administer mAbs to prevent progression to a severe form of COVID-19. According to national treatment guidelines for COVID-19, remdesivir is only indicated in patients with SpO_2_ < 93%. Also, in this case, remdesivir was not preferred given the possibility of aggravation of pre-existing arrhythmic events, as a number of cardiac adverse events with remdesivir in COVID-19 have already been reported in the literature [9]. At the same time, chronic colonization with MDR-*P. aeruginosa*, as well as numerous allergies and antimicrobial contraindications, would have made it difficult to use immunomodulators such as tocilizumab or anakinra or systemic corticosteroids in case the patient would have deteriorated and developed severe COVID-19. As previously shown, the use of IL-6 receptor antagonists in patients with severe COVID-19 is associated with a higher rate of bacterial superinfection [10].

CF is a complex multisystemic disease that primarily affects the lungs, and at this time still poses major treatment challenges. Any supplementary respiratory condition can have a severe course in these patients given the chronic microbial lung colonization or infection and the constant deterioration in functioning of the lungs [11]. However, the course of COVID-19 disease in patients with CF appears to be favorable [1,2,12]. Nevertheless, recent studies have shown that lung and pancreatic impairment are risk factors for severe COVID-19 in these patients [4,12]. Therefore, any CF patient with SARS-CoV-2 infection requires a comprehensive evaluation, taking into account all associated comorbidities, in order to tailor a personalized management plan.

SARS-CoV-2 mAbs are promising treatment options in patients with early-stage COVID-19. Data from clinical trials have shown high efficacy of mAbs in halting the progression to severe or critical COVID-19, preventing outcomes such as requirement of supplemental oxygen, intensive care admission or death. However, data from clinical trials on the use of mAbs in patients with multiple chronic conditions, including CF, are scarce. Thus, to our knowledge this is the first case report of a patient with CF and multiple comorbidities who was successfully treated with the monoclonal antibody cocktail, casirivimab and imdevimab.

## 4. Conclusions

We have reported the first administration of SARS-CoV-2 anti-spike mAbs in a patient with cystic fibrosis and moderate COVID-19, with good tolerability and good clinical evolution. As mAbs become part of routine clinical practice in Romania and elsewhere, it becomes essential to characterize the profile of patients who are expected to have the most benefit and the least risk from this type of treatment, and to prioritize timely diagnosis to allow early therapeutic intervention to prevent progression to severe disease.

## Figures and Tables

**Table 1:** Evolution of laboratory parameters during hospitalization.

Type of Laboratory Analysis	Date	2 Nov.	5 Nov.	11 Nov.	19 Nov.	23 Nov.
	Day of Illness	6	9	15	23	27
	Normal Range					
WBCs	5–12 × 10^3^/μL	10.32	8.04	9.64	-	-
Lymphocytes #	1.5–5.2 × 10^3^/μL	2.43	3.29	2.86	-	-
Lymphocytes %	32–48%	23.5	40.9	29.7	-	-
Neutrophils #	1.5–8.0 × 10^3^/μL	6.85	3.78	5.67	-	-
Neutrophils %	35–55%	**66.4**	47.1	**58.8**	-	-
Hemoglobin	13–15 g/dL	**17.7**	**16.5**	**16.1**	-	-
Platelets	150–450 × 10^3^/μL	231	248	292	-	-
C-reactive protein	<0.5 mg/dL	**1.23**	0.48	0.41	-	-
Fibrinogen	160–390 mg/dL	**432**	392	341	-	-
ESR	<15 mm/h	3	10	7	-	-
IL-6	0–7 pg/mL	**14.55**	2.51	2.42	-	-
AST	10–37 U/L	28	15	23	-	-
ALT	10–60 U/L	37	28	40	-	-
Urea	15–35 mg/dL	35	33	35	-	-
Creatinine	0.4–1.4 mg/dL	0.7	0.6	0.8	-	-
Ferritin	20–200 μg/L	120	149	-	-	-
D-dimer	0–0.5 mg/dL	0.2	0.2	0.3	-	-
IgM ^a^ (SARS-CoV-2)	-	negative	**positive**	-	**positive**	-
IgG ^a^ (SARS-CoV-2)	-	negative	negative	-	**positive**	-
IgM ^b^ (SARS-CoV-2)	positive > 1	0	**1.44**	-	**18.53**	-
IgG ^b^ (SARS-CoV-2)	positive > 1	0	0	-	1.81	-
RT-PCR SARS-CoV-2	-	**positive**	**positive**	**positive**	**positive**	**positive**

WBCs—white blood cells, ESR—erythrocyte sedimentation rate, IL-6—interleukin 6, AST—aspartate transaminase, ALT—alanine transaminase, IgM—immunoglobulin M, IgG—immunoglobulin G, RT-PCR—real-time polymerase chain reaction; #—absolute count; ^a^—rapid antibody test; ^b^—immunofluorescence assay—quantitative antibodies; In bold—abnormal lab values.

**Table 2:** Treatment recommendation at patient discharge.

Type of Drug	Name	Administration
Inhalation antibiotic therapy	Colistin	1,000,000 IU b.i.d.—wet nebulization
Mucolytic	Alfa-dornase	2500 IU q.d.—wet nebulization
	Hypertonic saline 3%	3 mL b.i.d.—wet nebulization
Bronchodilator	Ipratropium bromide 20 μg *	1 puff b.i.d.—inhaler
Enzyme replacement therapy	Pancreatin	10,000 UI/kg/day—orally
Fat-soluble vitamins	Vitamin A	3000 μg/day—orally
	Vitamin D	2000 IU/day—orally
	Vitamin E	200 mg/day—orally
	Vitamin K	200 μg/day—orally

* The patient had extrasystoles when taking salbutamol inhaler.

## Data Availability

Not applicable.

## Supplementary Materials

The following supporting information can be downloaded at: https://www.mdpi.com/article/10.3390/diagnostics12010137/s1, Video S1. Classic 3D rendering where fibrosis zones can be observed around the airways and the bronchiectasis, without architectural distortions. Video S2. Post-processed imagery using CT Lung Analyzer from Chest Imaging Platform where specific densities specific to infiltrated tissue were extracted (−750 HU–−450 HU); as presented, the most affected zones were the upper areas and perihilar area. Video S3. Post-processed imagery using CT Lung Analyzer from Chest Imaging Platform which shows the global overview of the subtracted densities, in which the infiltrated areas are observed (blue −950 HU–−750 HU). Video S4. Coronal analyzer: Automatically selected densities in the coronal plane in which the affected areas can be seen as follows: dark green in parenchyma—specific for emphysema (−1000 HU–−950 HU); blue—inflated specific densities (−950 HU–−750 HU); orange—infiltrated specific densities (−750 HU–−400 HU); purple—collapsed or more dense specific areas (−400 HU–−0 HU). HU—Hounsfield units.
